# Living Bacterial Microneedles for Fungal Infection Treatment

**DOI:** 10.34133/2020/2760594

**Published:** 2020-11-12

**Authors:** Fengyuan Wang, Xiaoxuan Zhang, Guopu Chen, Yuanjin Zhao

**Affiliations:** ^1^Department of Burns & Plastic Surgery, Institute of Translational Medicine, The Affiliated Drum Tower Hospital of Nanjing University Medical School, Nanjing 210008, China; ^2^Department of Dermatology, Zhongda Hospital, Southeast University, Nanjing 210009, China; ^3^State Key Laboratory of Bioelectronics, School of Biological Science and Medical Engineering, Southeast University, Nanjing 210096, China

## Abstract

Fungal infections are everlasting health challenges all over the world, bringing about great financial and medical burdens. Here, inspired by the natural competition law of beneficial bacteria against other microbes, we present novel living microneedles (LMNs) with functionalized bacteria encapsulation for efficient fungal infection treatment. The chosen beneficial bacterial components, *Bacillus subtilis* (*B. subtilis*), which are naturally found on the human skin and widely used for food processing, can get nutrients from the skin and escape from the immune system with the help of microneedles. Besides, the encapsulated *B. subtilis* can continuously produce and secrete various potential antifungal agents which can directly bind to fungal cell surface-associated proteins and destruct the cell membranes, thus avoiding drug resistance. After immobilization in the LMNs, the bacteria can stay within the LMNs without invasion and the encapsulated bacteria together with microneedles can be removed after application. Thus, the side effects, especially the risk for subsequent bacterial infections, are controlled to a minimum to ensure security. In addition, strong penetrability of the microneedles enhances penetration of antifungal agents, and their heights can be adjusted according to the infected depth to acquire better therapeutic effects. These features make the LMNs potentially valuable for clinical applications.

## 1. Introduction

Fungal infections, caused by various fungi such as *dermatophytes*, *Candida sp*, and *Malassezia sp*, may invade all kinds of tissues, organs, and implanted instruments in the human body and affect 20-25% of the population worldwide [1–3]. These fungal effects on human health are spiraling, and the global mortality rates of fungal diseases now exceed that of malaria or breast cancer [4, 5]. To eliminate fungal infections, many kinds of strategies have been developed, such as topical cream, oral antifungal drugs, suppository, and even surgery [6–10]. Among them, antifungal drugs such as azoles are the most typical and widespread frontline strategy used in humans [11, 12]. However, the fungal pathogen control is ephemeral by the antifungal drugs because the highly plastic genomes and rapid reproduction ability of fungi enable them to generate variants and quickly develop resistance to the chemicals [13, 14]. Besides, the antifungal drugs may cause lots of adverse drug reactions, including hepatotoxicity, gastrointestinal dysfunction, drug eruption, and anaphylaxis [15–17]. Additionally, although most of the antifungal drugs are lipophilic in nature, their high molecular mass leads to poor penetration and in turn causes low concentration achieved in cutaneous tissues, resulting in relapse of infections and frequent administration [18–20]. Therefore, effective antifungal therapy that can hinder emergence of resistance, minimize side effects, and enhance the penetration is still anticipated.

**Figure 1:**
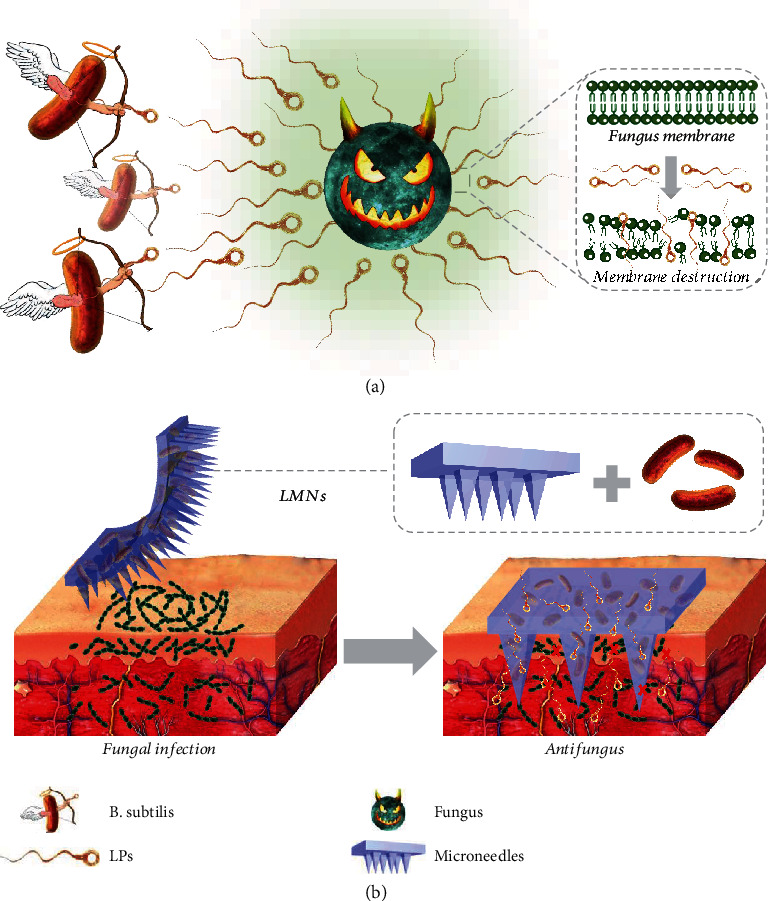
Schematic illustration of the application of the LMNs for fungal infection treatment. (a) The antifungal capability of the LPs released from *B. subtilis*. (b) The enhanced permeation of antifungal agents to the infected tissue directed by the microneedles.

In this paper, inspired by the natural competition law of beneficial bacteria and other microbes, we proposed novel living microneedles (LMNs) with functionalized bacteria encapsulation for fungal infection treatment, as schemed in Figure 1. In nature, the predominant bacteria can effectively repel, block, and interfere with other microbial reproduction and preempt living space through rapid, mass reproduction and colonization [21–23]. During this process, they could secrete abundant antimicrobial agents to enhance competitiveness [24, 25]. Thus, these beneficial bacteria have been observed with a wide range of bioactivities including antimicrobial, anticancer, immunosuppressive, and antimycoplasma [26–28]. However, the living bacterial therapy is often less than ideal due to uncontrollable bacterial sequelae, intrinsic bacterial toxicity, limited drug production, and contraindication with other therapies. In addition, it is challenging to colonize bacteria into the target site and protect them from the immune system to ensure effective therapeutic effects [29, 30]. Therefore, living bacteria still lack effective formulation and are rarely employed for practical treatment.

Here, we presented porous, localized, convenient, and painless hydrogel microneedles to realize living bacteria encapsulation and fungal infection treatment. The porous hydrogel microneedles were fabricated from photocured poly (ethylene glycol) diacrylate (PEGDA) and the sacrificed polyvinyl alcohol (PVA) component. The resultant porous microneedles could isolate and protect encapsulated living bacteria from the host immune system and continuously get nutrients from the skin to maintain bacterial activity [31–35]. Thus, by encapsulating a safe gram-positive bacteria *Bacillus subtilis* (*B. subtilis*) that are naturally found on the human skin and commercialized in the food industry, functional LMNs, which could efficiently produce and secrete a range of potential antifungal agents such as lipopeptides (LPs), directly extract fungal cell surface-associated proteins, destruct the cell walls, and kill the target fungi, were achieved. Through *in vitro* investigation, we have demonstrated that the bacteria were immobilized in the LMNs without invading outside while still exhibiting excellent antifungal capability. Besides, as the LMNs were fabricated flexibly, their height could be adjusted according to the depth of fungal infection, enhancing penetration of antifungal agents to the target site. In addition, the LMNs together with the encapsulated bacteria could be removed after application to avoid adverse effects caused by the left bacteria. Thus, the side effects of the LMNs were found to be controlled to a minimum, especially the occurrence and development of subsequent bacterial infections. These features make the LMNs valuable in practical fungal infection treatment and promising for various disease treatments.

## 2. Results

In a typical experiment, the porous microneedles were fabricated from an aqueous solution of biocompatible polymer poly (ethylene glycol) diacrylate (PEGDA) and polyvinyl alcohol (PVA) via a micromolding approach. The cross-linked PEGDA-based matrix could enhance the stiffness of the microneedles for efficient penetration through the skin, as well as enable sustained release of antifungal agents from the microneedles for maintaining high local concentrations in pathological tissues. In addition, the dispersive PVA component was sacrificed after contacting with body fluids and discharged with the metabolism of the epidermis, inducing pores in the photocured PEGDA hydrogel microneedles (Figure S1). The resultant porous microneedles could enhance nutrient transport from the skin to maintain bacterial activity. Furthermore, the thickness of the infected skin varies a lot due to different degrees of hyperkeratosis of the *stratum corneum*. To meet the demands of application in different skin parts, the microneedles with different base sizes and heights could be easily fabricated (Figure S2) by changing the parameters during the micromolding process. In this experiment, the selected microneedles were fabricated into a 15 mm by 15 mm patch with a 20 by 20 microneedle array, and each needle was pyramid-shaped with a base size of 230 *μ*m by 230 *μ*m and a height of 500 *μ*m.

**Figure 2:**
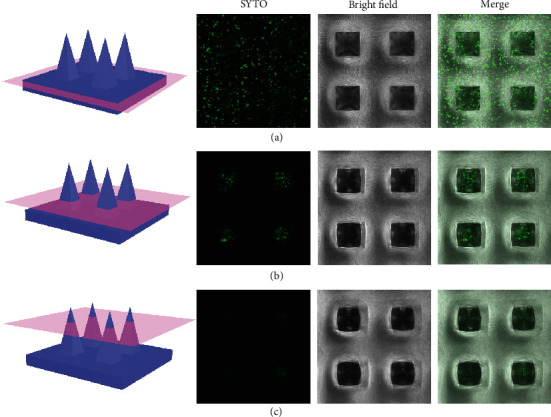
Viability and morphology of *B. subtilis* cultured in the microneedle patch. Immunofluorescent staining of the bottom (a), middle (b), and top (c) of the LMNs.

To fabricate LMNs, living bacteria were encapsulated in the PEGDA solution and then integrated into the microneedles. The gram-positive bacteria, *B. subtilis 3610*, were employed as the optimal bacterial component based on a tradeoff between their antifungal activity and the fact that they are nonmodified strains and the natural wild type. They are naturally found on the human skin, and their commercial strains have been marketed for the production of pharmaceutical proteins and for foodstuff processing. Such bacteria were uniformly dispersed in the PEGDA solution and solidified in the negative mold by ultraviolet (UV) light to fabricate LMNs. To evaluate activity of *B. subtilis* in the LMNs, immunofluorescence imaging of cross and lengthwise sections of the LMNs was conducted (Figure 2 and Figure S3). A large number of living bacteria stained with green fluorescence could be observed, indicating that *B. subtilis* could keep their viability after UV irradiation. Moreover, the integrity of the microneedles maintained well in phosphate-buffered saline (PBS, pH 7.4) after days 1, 3, and 7 (Figure S4), thus effectively avoiding the escape of bacteria brought about by the broken patch.

**Figure 3:**
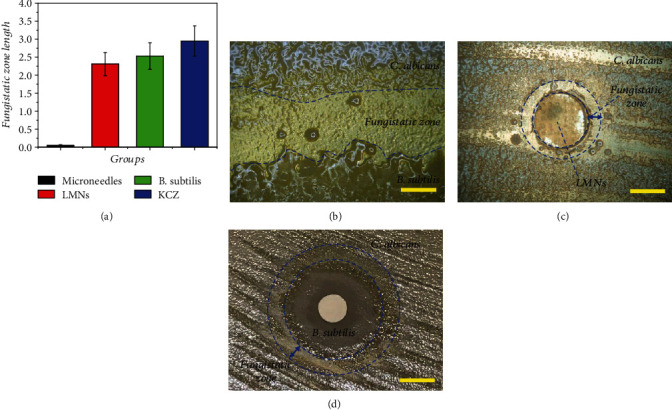
The *in vitro* antifungal capability of the LMNs. (a) Antifungal activity of microneedles, LMNs, *B. subtilis*, and KCZ against *C. albicans*. (b) Photo of the fungistatic zone formed between *B. subtilis* and *C. albicans.* (c) Fungistatic zone formed by the LMNs. (d) Fungistatic zone formed by the *B. subtilis* solution. Scale bar in (b) was 1.5 mm, in (c) was 3 mm, and in (d) was 8 mm.

To demonstrate the practical value of the LMNs for clinical applications, the *in vitro* antifungal capability of LMNs was evaluated using the disc diffusion method and was compared to that of empty microneedles, simple *B. subtilis* solution, and ketoconazole (KCZ) (Figure 3). A lawn of *C. albicans* was spread on yeast peptone dextrose agar medium at first. Afterwards, tested solutions (*B. subtilis* and ketoconazole) were added on paper filter discs (6 mm in diameter), while the LMNs and empty microneedles were directly cut into the same size and then placed at the center of each dish and incubated at 37°C for 48 h, respectively. The fungistatic zone could be obviously observed between the *B. subtilis* and *C. albicans* (Figure 3(b)). The LMNs demonstrated antifungal activity comparable to the KCZ, while the control microneedles did not exhibit antifungal activity (Figure 3(a)). It was worth nothing that the fungistatic zone width of the LMNs and *B. subtilis* solution was similar, but there was almost no *B. subtilis* found escaping from the LMNs while large amounts of the bacteria could be observed crawling out of the paper filter disc (Figures 3(c) and 3(d), Figure S5). This phenomenon indicated that the LMNs could play an antifungal role while avoiding the subsequent potential risk of bacterial infections.

The antifungal activity of *B. subtilis* against *C. albicans* could potentially be attributed to several factors. The natural secretion of LPs, especially surfactin, a most powerful biosurfactant that possesses a broad antifungal activity, is the key factor and has drawn wide attention recently. The mechanism of surfactin against *C. albicans* has been associated with extraction of cell surface-associated proteins and destruction of the cell wall. A liquid phase mass spectrometer confirmed the presence of surfactin in the LMNs, in accordance with the idea that surfactin played a constructive role in the exhibited antifungal activity (Figure S6 and S7). In addition to surfactin, which has been recognized as the most prominent surface-active biomolecule produced and secreted by *B. subtilis*, the bacteria could also secrete a lot of other LPs with strong antifungal properties, such as fengycin. In addition, the LMNs could penetrate the mouse skin at the dorsal site efficiently and release LPs controllably to both the surface and the deep part of the skin for drug permeation enhancement (Figure S8 and S9). The punctured sites could return to normal after a few minutes, indicating neglectable damage to the tissue (Figure S10).

**Figure 4:**
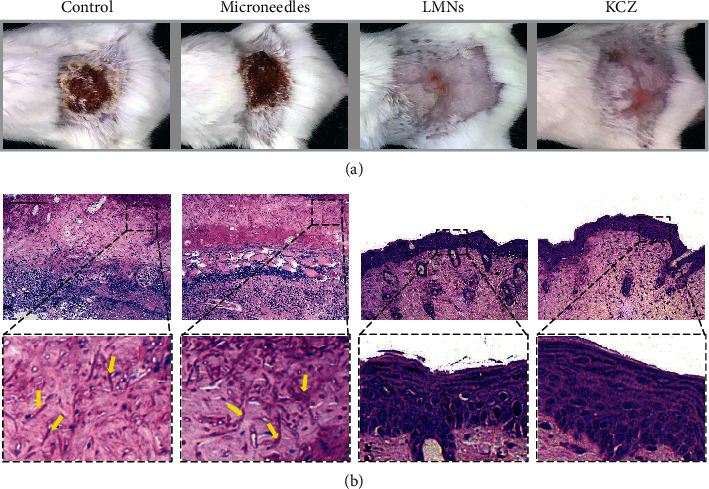
The *in vivo* antifungal capability of the LMNs. (a) Representative photos of the fungi-infected skin treated with PBS, microneedles, LMNs, and KCZ. (b) PAS staining of fungi-infected tissue after 14 d. Yellow arrows indicated the pseudohyphae of *C. albicans*. Scale bar in (b) was 100 *μ*m.

The efficacy of the LMNs was evaluated *in vivo* using a mouse cutaneous fungal infection model. Fungal infection was induced by smearing the 10^8^/ml conidia suspension of *C. albicans* on the areas of damaged dorsal skins in the immunosuppressed mice. Afterwards, mice were randomly assigned to control (phosphate-buffered saline (PBS)), microneedle, LMN, and KCZ groups. On the 14 d postinfection, there was a thick dark brown crust covering the inoculation sites in the control and microneedle groups. In contrast, there was no obvious infection and the fur started to grow again in the inoculation areas in both the LMN and KCZ groups (Figure 4(a)), indicating the less severe fungal infections.

**Figure 5:**
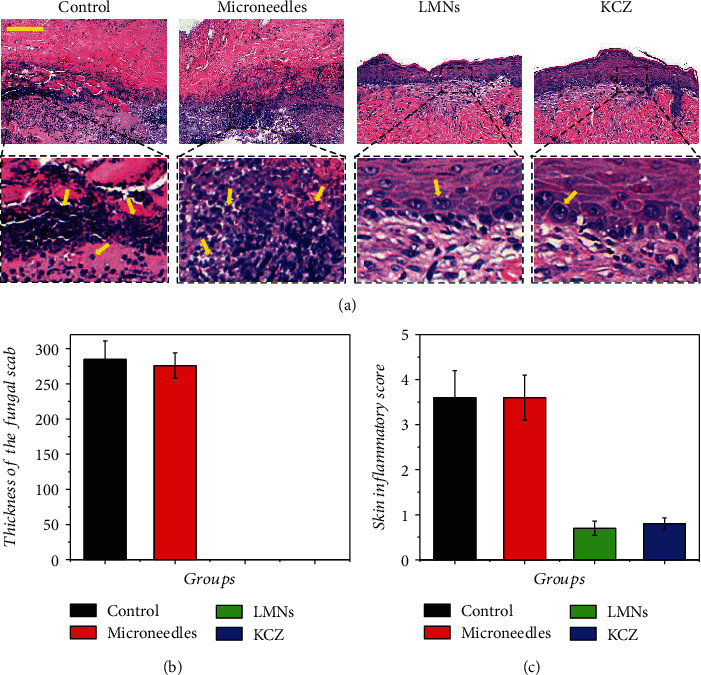
(a) HE staining of fungus-infected tissue after 14 d. (b) Quantification of thickness of the fungal scab. (c) Quantification of the skin inflammatory score. Yellow arrows indicated the inflammatory cells. Scale bar in (a) was 100 *μ*m.

To further investigate the biologic mechanism of the treating process, histopathology was performed to assess the exact situations of inflammation and infection. Periodic Acid-Schiff (PAS) staining was first performed to evaluate the fungal infection situation by observing pseudohyphae of the *C. albicans* (Figure 4(b)). The pseudohypha of *C. albicans* is the evading morphology of *C. albicans* in superficial infection and can be stained to be magenta. Large amounts of pseudohyphae could be observed in the control and microneedle groups as indicated with yellow arrows, showing the successful establishment of fungal infections and void treatments. On the contrary, there was no pseudohyphae found in the epidermis of the LMN and KCZ groups, indicating the successful antifungal effects. Furthermore, hematoxylin and eosin (HE) staining was exploited to evaluate the inflammation condition. In the control and microneedle groups, scabs composing of plenty of mononuclear and polymorphonuclear cells intermixed with fibroblastic proliferation formed in the epidermis (Figures 5(a) and 5(b)), indicating severe inflammatory response (Figures 5(a) and 5(c)). Adversely, in both of the LMN and KCZ groups, there were little inflammatory cells in the epidermis, suggesting minimal inflammatory response in the epidermis (Figures 5(a) and 5(c)). These results demonstrated that the LMNs could be effective in treating local fungal infections and other biomedical applications.

## 3. Discussion

In summary, we have presented living beneficial bacteria encapsulated microneedles for fungal infection treatment. The rapid emergence of multiresistant pathogenic fungi poses a considerable threat to disease control across the world. The first severe problem related to fungal infections is that fungi can resist drugs through target-site mutations, promoter changing, upregulation of efflux pumps, and activation of stress response pathways. Hence, it is urgent to develop novel antifungal agents with a new mechanism of action, disturb the emergence of resistance, and discover new disease control strategies to avoid overreliance on fungicides. Nature chooses the fittest to survive, and fungi are found to be naturally inhibited by some bacteria through spatial competition and release of antifungal agents, giving inspiration in dealing with fungal infection, whereas living bacteria employed for fungal infection treatment is seldom explored. Besides the drug resistance, fungal infections can also invade the dermis layer, fascia, mussel, and even bone, as well as all organs or implanted devices beyond the *stratum corneum*. Thus, to deal with this problem, several strategies including cream, paste, and oral administration are produced, whereas their therapeutic effects are limited by the poor permeation and lots of side effects. Therefore, more efforts are still anticipated to develop new therapeutic strategies with no resistance, minimal side effects, and high permeation.

Herein, we have fabricated localized, convenient, and painless microneedles with living bacteria encapsulation to solve the aforementioned problems. Through the bridge established between microneedles and tissues, the encapsulated *B. subtilis* could get nutrients from the skin to maintain activity and elude attack from the immune system. Afterwards, a range of potent antifungal agents were continuously produced by the bacteria and secreted to the infected tissues with no drug resistance. Owing to the inherent safety, the immobilization of LMNs, and the easy removal after application, the bacteria may cause minimal side effects. The strong penetrability and flexibility of the microneedles also imparted the LMNs with enhanced penetration of antifungal agents to target infected sites. The therapeutic effects of LMNs on skin fungal infections have also been proved using a mouse cutaneous fungal infection model. Apart from dealing with cutaneous fungal infections, the new strategies of LMNs are promising to be applied to organ fungal infections to overcome the limitations of oral administration caused by the liver first pass effect. Furthermore, due to the diversity of the bacterial functions, LMNs are also potential to be applied to treat cancer, bacterial infections, virus infections, etc. Thus, it is believed that the LMNs will be widely used in clinic based on their excellent capabilities.

## 4. Materials and Methods

### 4.1. Materials

Poly (ethylene glycol) diacrylate (PEGDA, average Mw = 700), 2-hydroxy-2-methylpropiophenone (HMPP), and Rhodamine B were purchased from Sigma-Aldrich Corp. The *B. subtilis 3610* and *C. albicans* (*SC5314*) were received from Bena Biotechnology Co. Ltd. Male BALB/c mice (6-8-week-old, 18-22 g) were treated in strict accordance with the Beijing Administration Rule of Animals in China, and all animal experiments in this study had received approval from School of Medicine, Southeast University.

### 4.2. Fabrication of Microneedles

Microneedles were fabricated by replicating uniform silicone molds purchased from WISECARE Corp (Taizhou, China). 250 *μ*l of PEGDA solution (50% *v*/*v*, dissolved in deionized water) mixed with PVA (10% *v*/*v*) and photoinitiator HMPP (1% *v*/*v*) was added to the mold and kept in a vacuum pump for 5 min to fully fill the cavities of the mold. The mixture was then cured by UV irradiation for 5 s under the UV-light system (EXFO OmniCure SERIES 1000, 365 nm, 100 W). Finally, the resultant MN arrays were gently peeled out of the mold. The size of the microneedle patch was 15 mm by 15 mm, with a 20 by 20 microneedle array, and each needle was pyramid-shaped with a base size of 230 *μ*m by 230 *μ*m and a height of 500 *μ*m. Notably, by changing different molds, microneedles with different base sizes, needle numbers, and heights could also be fabricated.

### 4.3. Fabrication of LMNs

A stock solution of *B. subtilis* which was maintained at -80°C was added to an LB agar petri dish. The plate was incubated in the incubator at 37°C overnight, and then, one colony was transferred into a glass tube containing 5 ml of fresh LB. The *B. subtilis* culture was taken between 3 and 6 h at 37°C. LMNs were fabricated with a similar procedure and formula to microneedles by using the PEGDA solution containing 4 × 10^8^/ml *B. subtilis in* LB as the material.

### 4.4. Characterization of LMNs

The different microneedles replicated from silicone molds were observed under a stereomicroscope (JSZ6S, Jiangnan Novel Optics) and captured by CCD (Oplenic digital camera). The integrity of the microneedles was recorded by immersing them in phosphate-buffered saline (PBS, pH 7.4) after days 1, 3, and 7. Surfactin extraction from *B. subtilis* was evaluated by a liquid phase mass spectrometer (LCMS6120, Agilent Ltd.). Living bacteria in the LMNs were stained by SYTO and observed by Opera Phenix (PerkinElmer Inc., UK).

### 4.5. Skin Penetration Test

The mice were anesthetized by intraperitoneal injection of 5% chloral hydrate. An electric clipper and depilatory cream were used to remove hair on the back of the skin. The microneedles were then inserted into the dorsal skin and remained tightly pressed for 1 min. The dorsal skin was observed by using a stereomicroscope (JSZ6S, Jiangnan Novel Optics) until the insertion marks disappeared completely. Rhodamine B was employed to simulate drugs and was encapsulated in the microneedles. Rhodamine B-laden microneedles were inserted into the dorsal skin, and the drug permeation in the skin was observed by Opera Phenix (PerkinElmer Inc., UK).

### 4.6. Antifungal Activity *In Vitro*

The fungus was cultured on the yeast peptone dextrose agar medium for 48 h, and the conidia were collected by PBS. The concentration of the conidia suspension was diluted to 10^8^/ml, and a volume of 50 *μ*l of the 10^8^/ml conidia suspension was spread on the yeast peptone dextrose agar plate. The discs (6 mm in diameter) loaded with 10^8^*B. subtilis* and 10 *μ*g ketoconazole, respectively, and empty microneedles and LMNs with 10^8^*B. subtilis* were put onto the plate. The microneedles were cut into circles with the same diameter of discs. The widths of the fungistatic zone were measured after 48 h incubation at 37°C.

### 4.7. Antifungal Effect *In Vivo*

The murine model of fungal infections was established to evaluate the *in vivo* antifungal effect of LMNs. The establishment of the model started with the induction of immunosuppression. Cyclophosphamide was injected intraperitoneally by 150 mg/kg of body weight on days 3 and 1 before the infection, and hydrocortisone acetate was injected subcutaneously by 40 mg/kg of body weight on day 1 before the infection to induce an immunosuppression state and increase the infected rate. The immunosuppression state was maintained by injecting cyclophosphamide by 75 mg/kg of body weight every 3 days. The mice were anesthetized with isoflurane, and their back was shaved for a 2cm∗2cm square. The exposed skins were rubbed with abrasive paper until the blood seeped out. The infection was caused by smearing the 10^8^/ml conidia suspension on the areas of damaged skins three times with sterile swabs. Inoculation of the fungus to a moist, warm, and blood-rich local wound could quickly result in the formation of superficial fungal infection lesions within 1 day.

The treatment started on day 1 postinfection. The mice were once a day treated separately with 200 *μ*l of sterile PBS for control, 25 mg of 2% ketoconazole cream (containing 0.5 mg ketoconazole), microneedles, and LMNs (containing 10^8^*B. subtilis*). Considering the similar therapeutic effects and less damage to the skin tissue, microneedles with a height of 500 *μ*m were chosen. The mice were sacrificed on day 14 postinfection, and the infected skin tissues were removed and put into 4% paraformaldehyde overnight in preparation for histological analysis. The histological tissues were stained with hematoxylin and eosin staining (HE staining) and Periodic Acid-Schiff staining (PAS staining).

## Data Availability

All data needed to evaluate the conclusions in the paper are present in the paper. Additional data related to this paper may be requested from the authors.

## Supplementary Materials

Supplementary Materials Figure S1: characterization of the porous microneedles. Figure S2: fabrication and characterization of microneedles with different sizes. Figure S3: immunofluorescence staining of living bacteria in the LMNs. Figure S4: degradation of the microneedles immersed in PBS. Figure S5: amount of the B. subtilis escaping from LMNs after immersing in PBS. Figure S6: MS/MS spectra of C12 surfactin homologue *m*/*z* 994. Figure S7: MS/MS spectra of C16 surfactin homologue *m*/*z* 1050. Figure S8: HE staining of the skin penetrated by the microneedles. The black arrow indicated the penetrating site. Figure S9: Rhodamine B staining macrograph and fluorescent image of mouse skin administered with microneedles. Figure S10: images of dorsal skin after inserting the microneedles at 0 min and 20 min.

## References

[1] Fisher M. C., Hawkins N. J., Sanglard D., Gurr S. J. (2018). Worldwide emergence of resistance to antifungal drugs challenges human health and food security. *Science*.

[2] Cornely O. A., Alastruey-Izquierdo A., Arenz D. (2019). Global guideline for the diagnosis and management of mucormycosis: an initiative of the European Confederation of Medical Mycology in cooperation with the Mycoses Study Group Education and Research Consortium. *The Lancet Infectious Diseases*.

[3] Naglik J. R., Gaffen S. L., Hube B. (2019). Candidalysin: discovery and function in Candida albicans infections. *Current Opinion in Microbiology*.

[4] Fisher M. C., Henk D. A., Briggs C. J. (2012). Emerging fungal threats to animal, plant and ecosystem health. *Nature*.

[5] Zhang C., Yang M., Ericsson A. C. (2019). Antimicrobial peptides: potential application in liver cancer. *Frontiers in Microbiology*.

[6] Zhang Y., Tu J., Wang D. (2018). Programmable and multifunctional DNA-based materials for biomedical applications. *Advanced Materials*.

[7] Gupta S. K., Li Y., Guo M. (2019). Anisotropic mechanics and dynamics of a living mammalian cytoplasm. *Soft Matter*.

[8] Li J., Cha R., Mou K. (2018). Nanocellulose-based antibacterial materials. *Advanced Healthcare Materials*.

[9] Ying G. L., Jiang N., Maharjan S. (2018). Aqueous two-phase emulsion bioink-enabled 3D bioprinting of porous hydrogels. *Advanced Materials*.

[10] Zhang Z., Huang X., Qian Y., Chen W., Wen L., Jiang L. (2019). Engineering smart nanofluidic systems for artificial ion channels and ion pumps: from single-pore to multichannel membranes. *Advanced Materials*.

[11] Fisher B. T., Zaoutis T., Dvorak C. C. (2019). Effect of caspofungin vs fluconazole prophylaxis on invasive fungal disease among children and young adults with acute myeloid leukemia: a randomized clinical trial. *JAMA*.

[12] Demuyser L., Van Dijck P. (2019). Can Saccharomyces cerevisiae keep up as a model system in fungal azole susceptibility research?. *Drug Resistance Updates*.

[13] Demers E. G., Biermann A. R., Masonjones S. (2018). Evolution of drug resistance in an antifungal-naive chronicCandida lusitaniaeinfection. *Proceedings of the National Academy of Sciences of the United States of America*.

[14] Gao J., Wang H., Li Z. (2018). Candida albicans gains azole resistance by altering sphingolipid composition. *Nature Communications*.

[15] Kishimoto T. K., Ferrari J. D., LaMothe R. A. (2016). Improving the efficacy and safety of biologic drugs with tolerogenic nanoparticles. *Nature Nanotechnology*.

[16] Lufton M., Bustan O., Eylon B. H. (2018). Living bacteria in thermoresponsive gel for treating fungal infections. *Advanced Functional Materials*.

[17] Yang X., Zhang L., Jiang X. (2018). Aminosaccharide–gold nanoparticle assemblies as narrow-spectrum antibiotics against methicillin-resistant Staphylococcus aureus. *Nano Research*.

[18] Younes N. F., Abdel-Halim S. A., Elassasy A. I. (2018). Solutol HS15 based binary mixed micelles with penetration enhancers for augmented corneal delivery of sertaconazole nitrate: optimization, in vitro, ex vivo and in vivo characterization. *Drug Delivery*.

[19] Strydom N., Gupta S. V., Fox W. S. (2019). Tuberculosis drugs’ distribution and emergence of resistance in patient’s lung lesions: a mechanistic model and tool for regimen and dose optimization. *PLoS Medicine*.

[20] Zhang K., Jia Z., Yang B. (2018). Adaptable hydrogels mediate cofactor-assisted activation of biomarker-responsive drug delivery via positive feedback for enhanced tissue regeneration. *Advancement of Science*.

[21] Itoh H., Jang S., Takeshita K. (2019). Host–symbiont specificity determined by microbe–microbe competition in an insect gut. *Proceedings of the National Academy of Sciences of the United States of America*.

[22] Patnode M. L., Beller Z. W., Han N. D. (2019). Interspecies competition impacts targeted manipulation of human gut bacteria by fiber-derived glycans. *Cell*.

[23] Raffatellu M. (2018). Learning from bacterial competition in the host to develop antimicrobials. *Nature Medicine*.

[24] Piewngam P., Zheng Y., Nguyen T. H. (2018). Pathogen elimination by probiotic Bacillus via signalling interference. *Nature*.

[25] Bueso Y. F., Lehouritis P., Tangney M. (2018). In situ biomolecule production by bacteria; a synthetic biology approach to medicine. *Journal of Controlled Release*.

[26] Helmink B. A., Khan M. W., Hermann A., Gopalakrishnan V., Wargo J. A. (2019). The microbiome, cancer, and cancer therapy. *Nature Medicine*.

[27] Chowdhury S., Castro S., Coker C., Hinchliffe T. E., Arpaia N., Danino T. (2019). Programmable bacteria induce durable tumor regression and systemic antitumor immunity. *Nature Medicine*.

[28] Chen G., Yu Y., Wu X. (2019). Microfluidic electrospray niacin metal-organic frameworks encapsulated microcapsules for wound healing. *Research*.

[29] Hao Y., Li Y., Zhang F. (2018). Electrochemical responsive superhydrophilic surfaces of polythiophene derivatives towards cell capture and release. *ChemPhysChem*.

[30] Shao C., Liu Y., Chi J., Wang J., Zhao Z., Zhao Y. (2019). Responsive inverse opal scaffolds with biomimetic enrichment capability for cell culture. *Research*.

[31] Tang J., Wang J., Huang K. (2018). Cardiac cell–integrated microneedle patch for treating myocardial infarction. *Science Advances*.

[32] Ye Y., Yu J., Wang C. (2016). Microneedles integrated with pancreatic cells and synthetic glucose-signal amplifiers for smart insulin delivery. *Advanced Materials*.

[33] Zhang X., Wang F., Yu Y. (2019). Bio-inspired clamping microneedle arrays from flexible ferrofluid-configured moldings. *Scientific Bulletin*.

[34] Zhang X., Sun L., Wang Y., Bian F., Wang Y., Zhao Y. (2019). Multibioinspired slippery surfaces with wettable bump arrays for droplets pumping. *Proceedings of the National Academy of Sciences of the United States of America*.

[35] Zhang X., Chen G., Bian F., Cai L., Zhao Y. (2019). Encoded microneedle arrays for detection of skin interstitial fluid biomarkers. *Advanced Materials*.

