# Supplementary material for: Living Bacterial Microneedles for Fungal Infection Treatment
Source: Research (Wash D C). 2020 Nov 12;2020:2760594. doi: 10.34133/2020/2760594 (PMC7877375; doi:10.34133/2020/2760594)
Supplement: Supplementary Materials — Figure S1: characterization of the porous microneedles. Figure S2: fabrication and characterization of microneedles with different sizes. Figure S3: immunofluorescence staining of living bacteria in the LMNs. Figure S4: degradation of the microneedles immersed in PBS. Figure S5: amount of the B. subtilis escaping from LMNs after immersing in PBS. Figure S6: MS/MS spectra of C12 surfactin homologue m/z 994. Figure S7: MS/MS spectra of C16 surfactin homologue m/z 1050. Figure S8: HE staining of the skin penetrated by the microneedles. The black arrow indicated the penetrating site. Figure S9: Rhodamine B staining macrograph and fluorescent image of mouse skin administered with microneedles. Figure S10: images of dorsal skin after inserting the microneedles at 0 min and 20 min. [file 2760594.f1.docx]

Supplementary Materials for

**Living bacterial microneedles for fungal infections treatment**

Fengyuan Wang, Xiaoxuan Zhang, Guopu Chen, Yuanjin Zhao*

*Corresponding author. Email: yjzhao@seu.edu.cn

**Supplemental Figures**


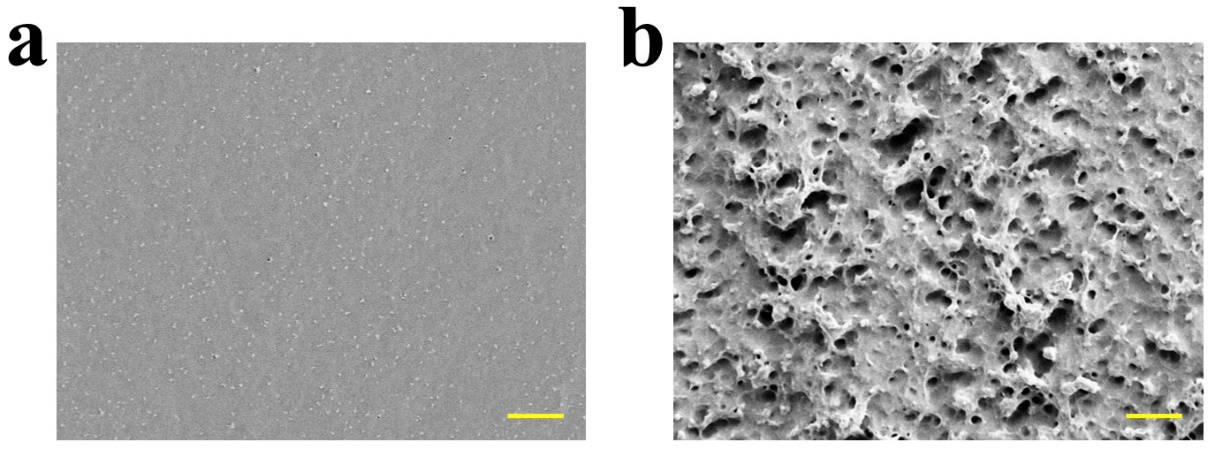


**Figure S1.** **Characterization of the porous microneedles.** Scanning electron microscope (SEM) of (a) simple PEGDA hydrogel microneedles and (b) porous microneedles.

Scar bars in (a, b) were 1 μm.


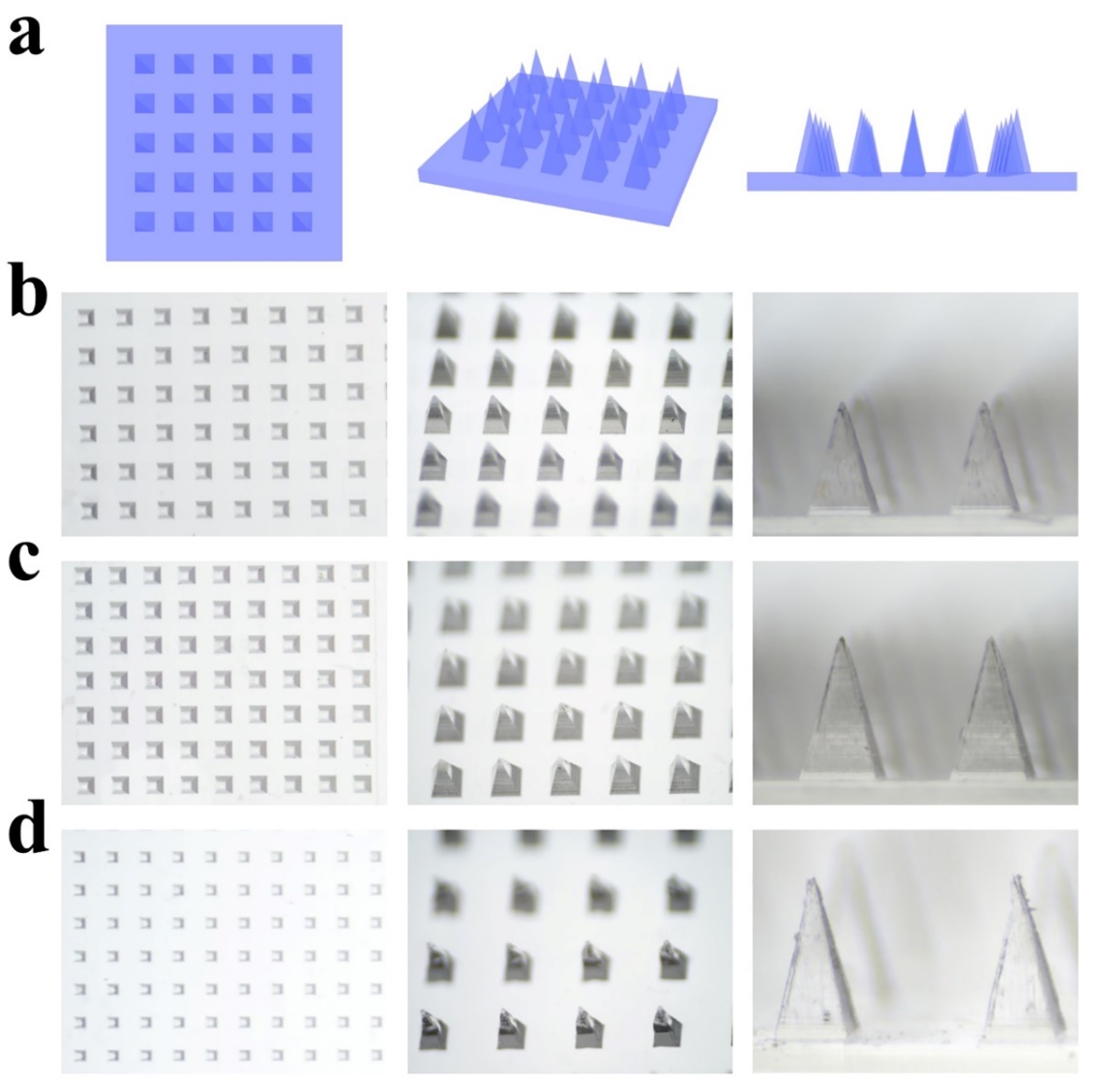


**Figure S2.** **Fabrication and characterization of microneedles with different sizes.** (a) Schematic illustration of the shooting angle. (b-c) Optical images of microneedles

with the height of (b) 500 μm, (c) 600 μm, and (d) 800 μm.


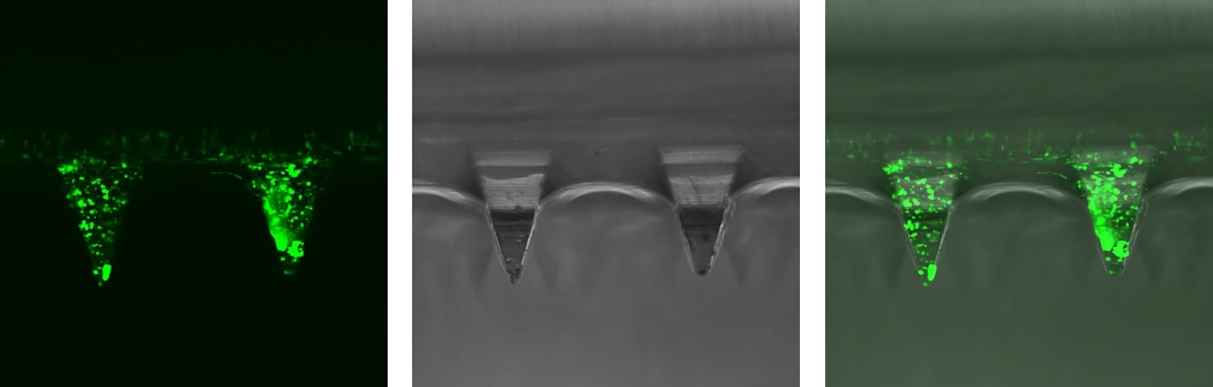


**Figure S3.** Immunofluorescence staining of living bacteria in the LMNs.


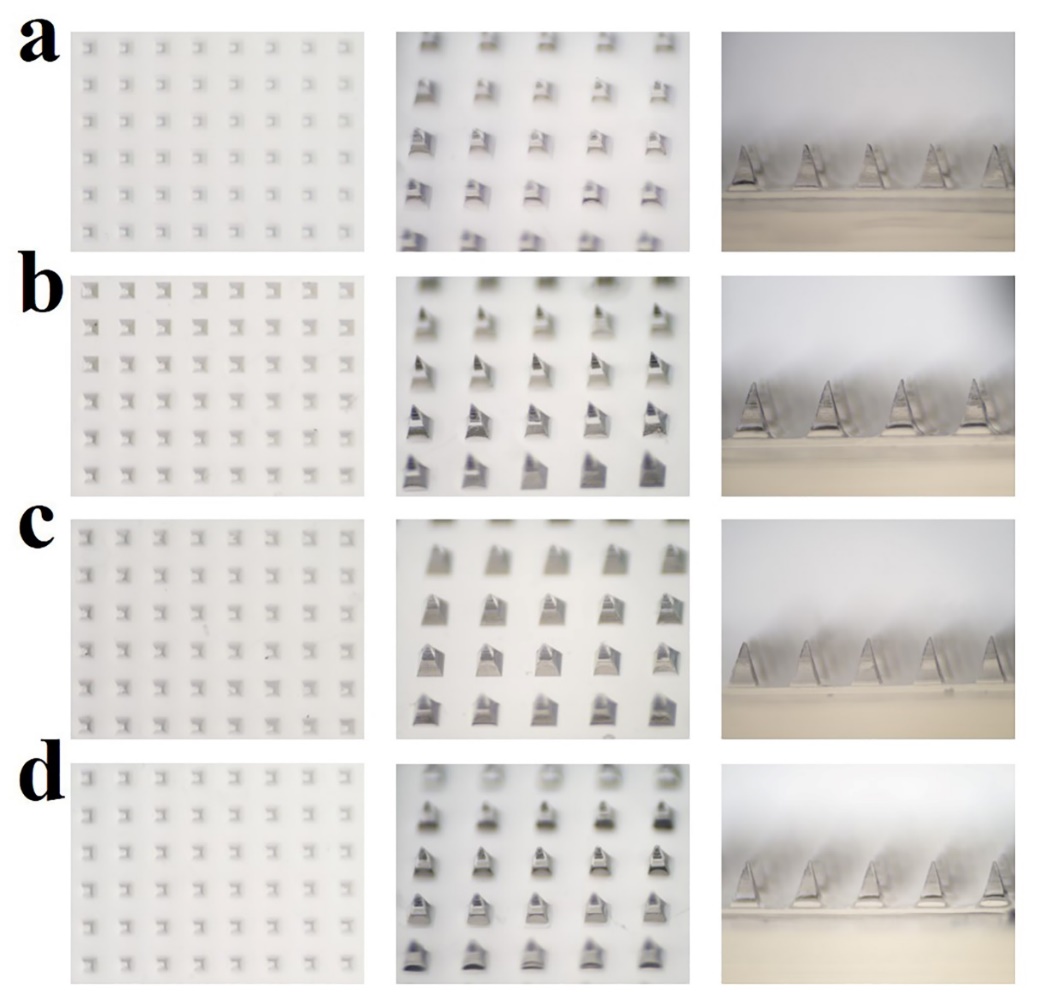


**Figure S4.** Degradation of the microneedles immersed in PBS after (a) 1d, (b) 3 d, (c) 5 d,

and (d) 7 d.


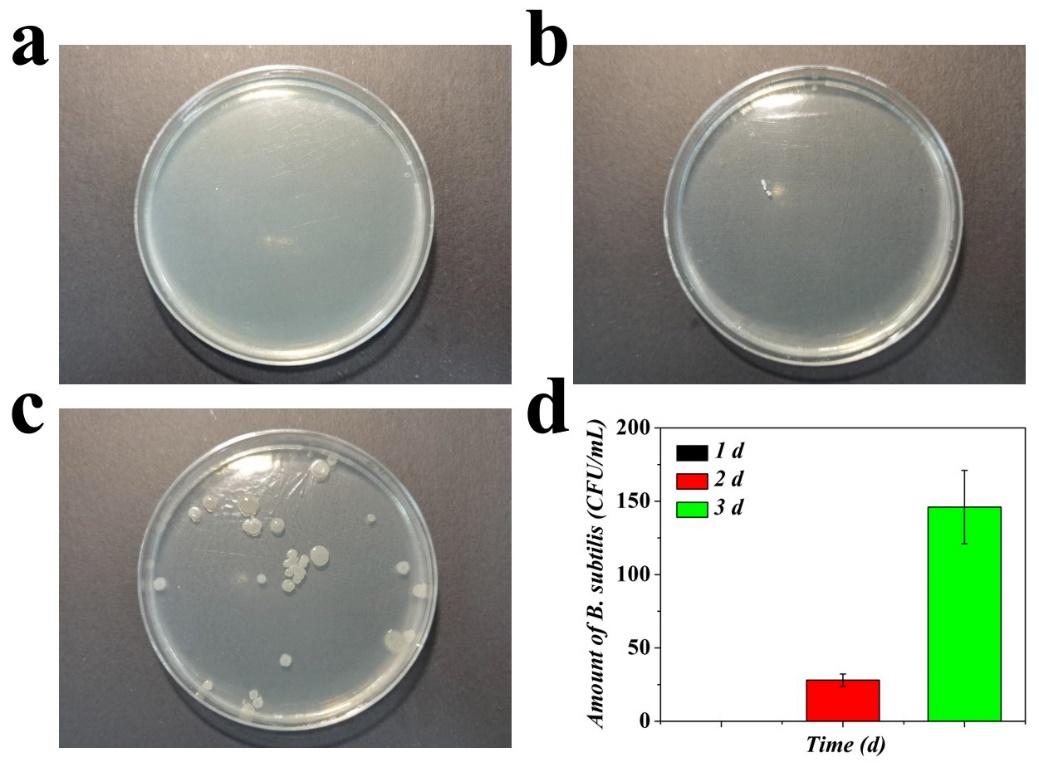


**Figure S5.** Amount of the *B. subtilis* escaped from LMNs after immersing in PBS for (a)1d, (b)2d, and (c) 3d. (d) Quantification of amount of *B. subtilis* in the PBS.


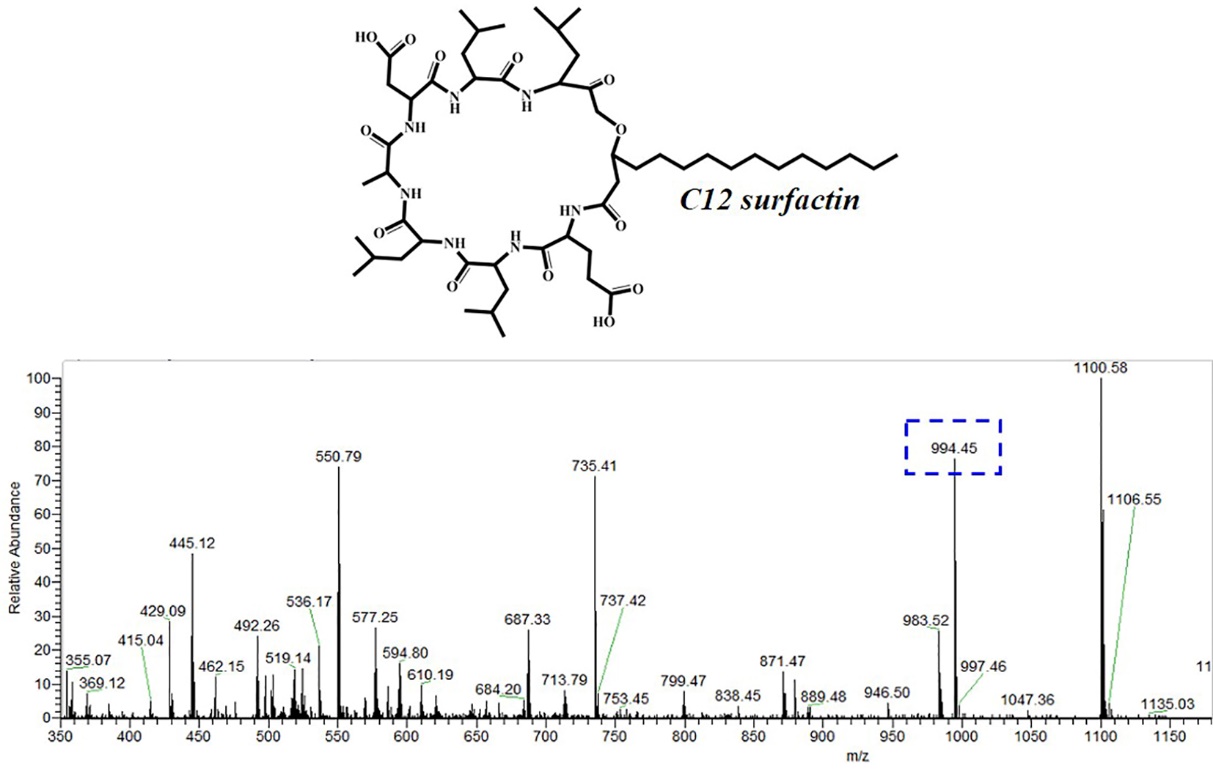


**Figure S6.** MS/MS spectra of C12 surfactin homologue m/z 994.


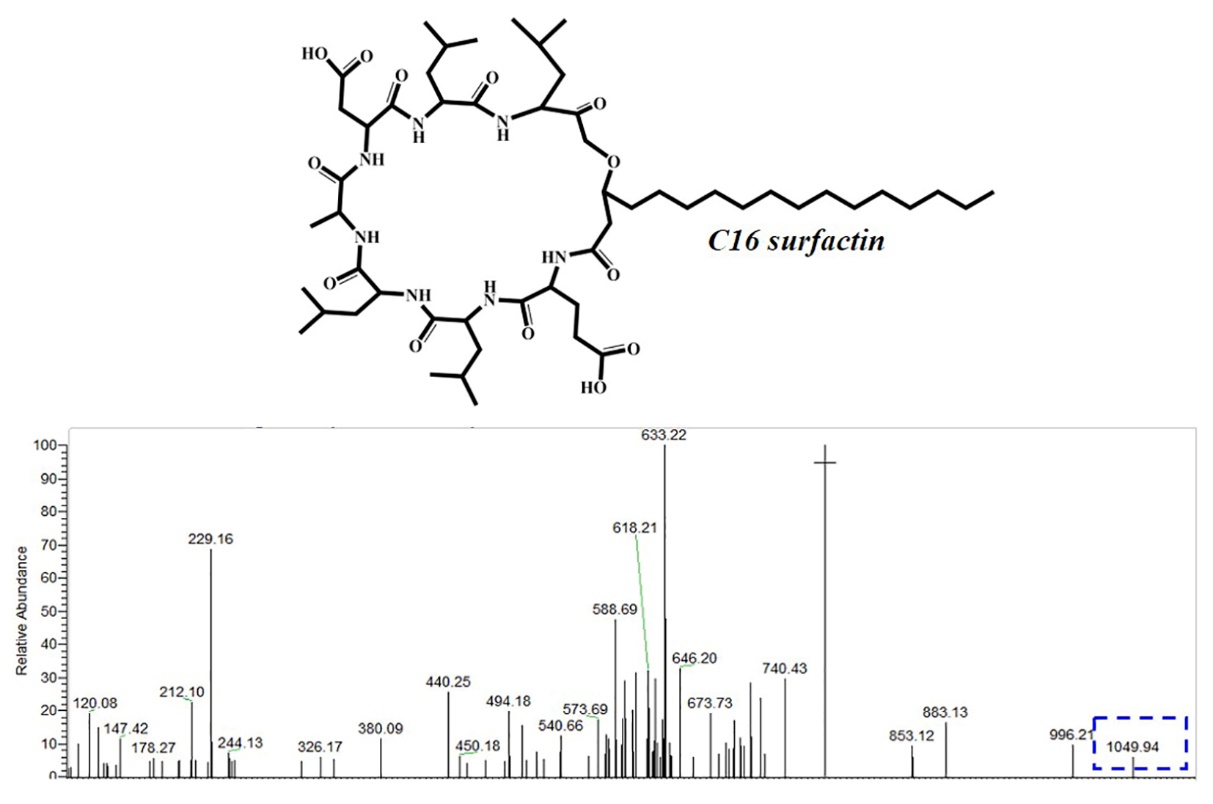


**Figure S7.** MS/MS spectra of C16 surfactin homologue m/z 1050.


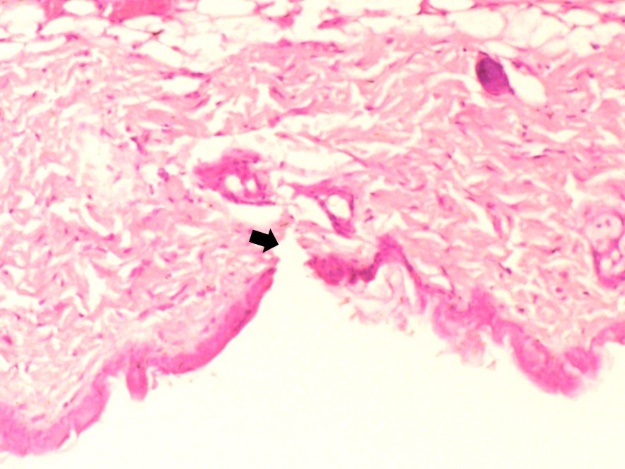


**Figure S8.** HE staining of the skin penetrated by the microneedles. The black arrow indicated the penetrating site.


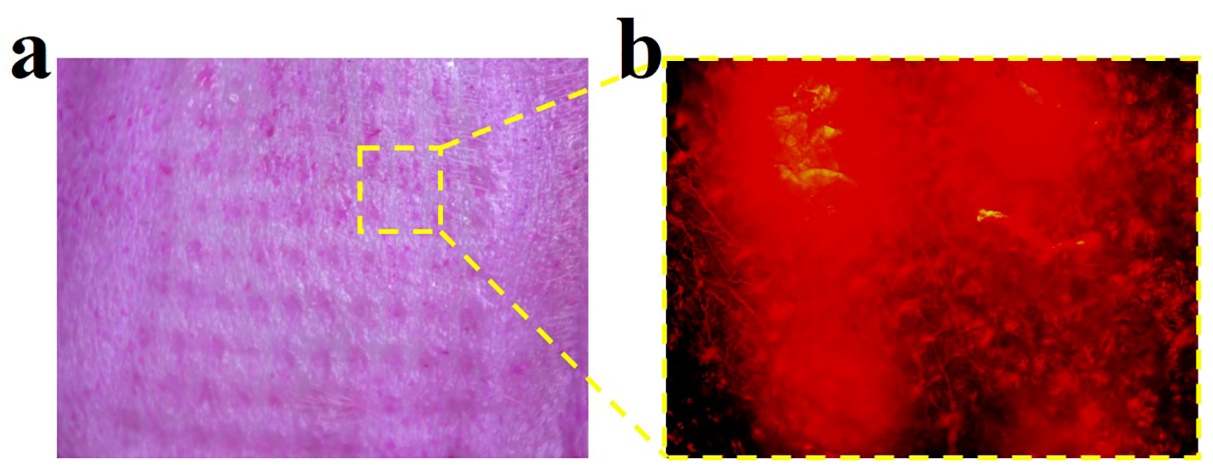


**Figure S9.** Rhodamine B staining (a) macrograph and (b) fluorescent image of mouse skin administered with microneedles.


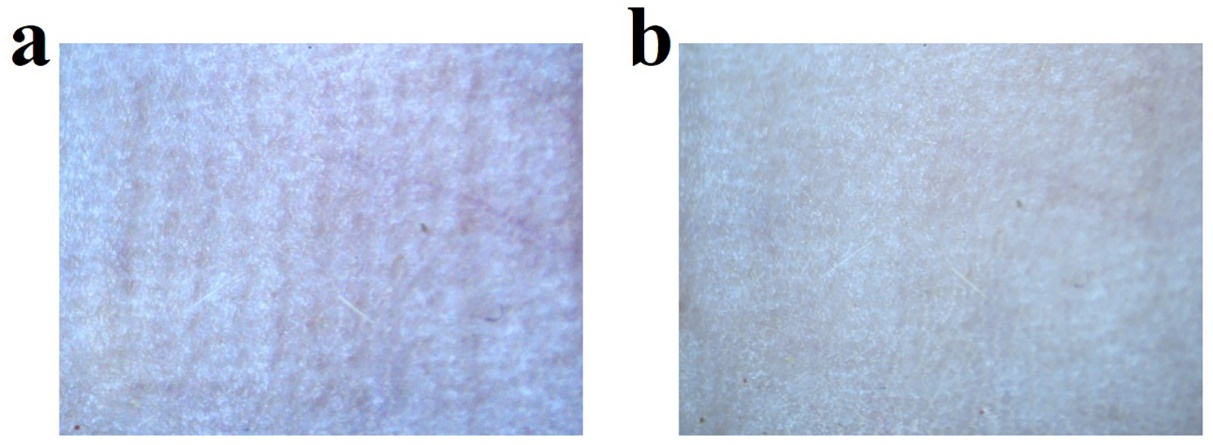


**Figure S10.** Images of dorsal skin after inserting the microneedles at (a) 0 min and (b) 20 min.
